# Retraction: Tbx1 Regulates the BMP-Smad1 Pathway in a Transcription Independent Manner

**DOI:** 10.1371/journal.pone.0230974

**Published:** 2020-03-19

**Authors:** The PLOS ONE Editors

Following the publication of this article [1], concerns have been raised regarding Figs 1, 2, and 3.

- Discrepancies have been detected in the background of both lanes presented in Fig 1B, particularly around regions marked as 70kDa and 20kDa. The underlying data to support Fig 1B are not available.
- Horizontal and vertical discrepancies have been detected in the background surrounding lane 3 of Fig 2A. The corresponding author has indicated that the figure was prepared using immunostaining from two different filters. The underlying data of lane 1 and lane 2 support the published figure, the underlying data to support lane 3 are no longer available.
- In Fig 2B, panels IP:anti-Flag Detect: anti-c-myc and IP: anti-c-myc Detect: anti-Flag, there are apparent smears in the background around lane 4 of the IP:anti-Flag Detect: anti-c-myc panel, and around lane 3 of the IP: anti-c-myc Detect: anti-Flag panel. The scans of the underlying blots at different exposures confirm the smears were present on the original blot.
- The bands in lanes 3 and 4 of the WB: anti-Smad4 panel of Fig 3C appear similar. The original blot underlying Fig 3C is not available, and a previous scan of the underlying data does not match the published panel.

In light of the unresolved concerns that question the integrity of these data, the *PLOS ONE* Editors retract this article.

FGF, TH, and PJS agreed with the retraction. AB agreed with the retraction based on the anomalies in Fig 1B, and apologises for not having noticed the anomalies earlier. PJS and AB stand by the conclusions of the article.

## References

[1] Fulcoli FG, Huynh T, Scambler PJ, Baldini A (2009) Tbx1 Regulates the BMP-Smad1 Pathway in a Transcription Independent Manner. PLoS ONE 4(6): e6049 10.1371/journal.pone.0006049 19557177 PMC2698216

